# Do not forget the boys – gender differences in children living in high HIV-affected communities in South Africa and Malawi in a longitudinal, community-based study

**DOI:** 10.1080/09540121.2016.1176680

**Published:** 2016-07-08

**Authors:** I. S. Hensels, L. Sherr, S. Skeen, A. Macedo, K. J. Roberts, M. Tomlinson

**Affiliations:** ^a^ Department of Infection and Population Health, University College London, London, UK; ^b^ Department of Psychology, Stellenbosch University, Stellenbosch, South Africa; ^c^ Department of Psychiatry and Mental Health, University of Cape Town, Cape Town, South Africa

**Keywords:** Gender, HIV/AIDS, children, community, education, violence

## Abstract

Gender is an important factor in child development. Especially in sub-Saharan Africa, girls have often been shown to be less likely to access education compared to boys. The consequence of this has been that that programmes addressing child development are often aimed at girls in order to redress gender imbalances. This study examines the effect of gender on the development of children attending community-based organisations in high HIV-affected areas, and explores whether community-based organisation attendance was associated with any changes in gender differences over time. Baseline data from 989 children and 12–15 month follow from 854 (86% response rate) were used to examine gender differences in children from Malawi and South Africa. At baseline, where there were differences by gender, these tended to disadvantage boys. It was found that boys were significantly more often found to be subjected to violence. Boys showed worse performance at school and more behavioural problems than girls. These gender differences persisted from baseline to follow-up. At follow-up, boys self-reported significantly worse average quality of life than girls. Only harsh discipline differed by gender in progression over time: boys experienced a stronger reduction in harsh physical discipline than girls from baseline to follow-up. Since harsh discipline was associated with boys’ worse educational outcomes and behavioural problems, our data cautiously suggests that gender differences could be reduced over time. In conclusion, our data suggests that, perhaps due to the narrow equity approach focusing on provision for girls, boys may be overlooked. As a result, there are some specific experiences where boys are generally worse off. These differences have distinct ramifications for the educational and emotional development of boys. A broader equity approach to child development might be warranted to ensure that the needs of both girls and boys are considered, and that boys are not overlooked.

## Introduction

Gender is an important driver of child outcomes (Park & Brondi, 2015). Gender differences have been found in in educational outcomes, cognition, language (Wallace et al., 2015), physical growth (Wamani, Astrøm, Peterson, Tumwine, & Tylleskär, 2007), socialisation (Rubin & Barstead, 2014) and parental interactions (Barbu et al., 2015; Park & Brondi, 2015). Thus, gender differences have been well documented, but all too often studies on children conflate gender and thereby hide or overlook any gender-specific findings which can guide differentiation in response and provision (Sherr, Mueller, & Varrall, 2009). Gender roles may impede progress and opportunity in a society which is “rife with gender stereotypes and biases” (Witt, 1997, p. 1).

In the fight against HIV infection and AIDS, gender is a driving factor. Gender differences have been noted in both the biological response to HIV and social responses (Bautista-Arredondo, Servan-Mori, Beynon, González, & Volkow, 2015; Idele et al., 2014). Gender discrimination can affect equity of provision (Bhana, 2007) and social roles, as well as role differentiation may affect life course outcomes (Gahagan, Gray, & Whynacht, 2015). Yet, while many adult-related HIV studies have a strong gender focus, the same cannot be said for children. For example, current UNAIDS data still collect numbers of children without providing gender breakdown until adolescence and adulthood (UNAIDS, 2015).

HIV has challenged children and their living circumstances. Illness, poverty, bereavement, deprivation, stigma and hardship have been well documented for both infected and affected children (Cluver, Gardner, & Operario, 2007; Francis-Chizororo, 2010; Sherr et al., 2014). Gender issues for these children need to be explored and incorporated in response and provision (Heidari et al., 2012; Sherr et al., 2009). A recent six Caribbean country study by Peltzer and Pengpid (2015) showed that early sexual debut among boys was significantly higher than that among girls (37.2% versus 16.9%). This disparity was confirmed in an eight-country study across Africa which further showed that the associations and predictors differed by gender (Peltzer, 2010). A study looking at children’s representations of HIV-affected peers showed that boys were depicted as suffering greater hardships. The authors question whether this represents greater challenges faced by boys or highlights gender inequality where female suffering is seen as less bad than male suffering (LeRoux-Rutledge et al., 2015). There are particular negative effects on female children, most notably in terms of educational opportunity, household burden and caregiving burden (Knight & Yamin, 2015; Thurman, Kidman, Nice, & Ikamari, 2015). Gender role allocation may have affected a number of outcomes. For example, many studies on young children and HIV have focused mostly on mothers without the inclusion of fathers, yet paternal inclusion is advantageous (Auvinen, Kylmä, & Suominen, 2013; Ditekemena et al., 2012; Sherr & Croome, 2012). On the other hand, many studies have shown disadvantages for girls and as a result, a number of initiatives to provide opportunities for girls have evolved (Beattie et al., 2015; Hardee, Gay, Croce-Galis, & Afari-Dwamena, 2014; Hardee, Gay, Croce-Galis, & Peltz, 2014). In such an era, it is important to understand the needs and under-provision for both girls and boys. A strong case has been made for the equality needs of girls; yet, it is important to ensure that a focus on gender disparity does not inadvertently disadvantage boys – equality at the cost of equity.

Given the importance of gender, and the need for both equality and equity of provision, this study examined the variables within the Child Community Care study, a multi-country study on children affected by HIV, according to gender. The specific aim of the study was to provide an understanding of gender effects on functioning for young children (aged 4–13 years) in relation to health, education, parenting, mental health and psychological parameters within the study population. Such data would be key in providing guidance and insight into future directions for policy and services.

## Methods

### Sample selection

Children between the ages of 4 and 13 years were recruited from community-based organisations in South Africa and Malawi. These countries were selected for their high HIV prevalence. Community Based Organisation’s (CBOs) provided community-based psychosocial support and services to children and families infected and affected by HIV. The type of community-based programme services the children received was based on carer report. Most children received food provision (*n* = 537, 54.3%) and play supervision (*n* = 498, 50.4%). Other services included home-based care (*n* = 262, 26.5%), early child development programmes (*n* = 259, 26.2%), psychosocial interventions (*n* = 252, 25.5%), access to clothes and blankets (*n* = 246, 24.9%), educational services (*n* = 243, 24.6%), emotional support (*n* = 166, 16.8%) and social grants (*n* = 116, 11.7%). Fewer children received skills-building training (*n* = 96, 9.7%), medical provision (*n* = 69, 7%), emergency services (*n* = 41, 4.1%) and income support directly to children and families (*n* = 6, 0.6%); yet those in need received assistance to access these services, for example through referrals (*n* = 91, 9.2%). Carers also reported on the types of services they received from the CBO they attended. The majority received child support interventions (*n* = 644, 65.1%) and material supplies (*n* = 401, 40.5%). Other services included parenting interventions (*n* = 297, 30%), home visits (*n* = 262, 26.5%), education services (*n* = 243, 24.6%), assistance in accessing grants (*n* = 116, 11.7%) and medical services (*n* = 102, 10.3%). Eleven funding partners (World Vision, Comic Relief, Save the Children, Firelight Foundation, Help Age, UNICEF, REPSSI, Bernard van Leer Foundation, STOP AIDS Now, AIDS Alliance and the Diana Memorial Fund) participated in the study to provide access to the CBOs. All 588 of their funded community-based organisations that provided services to children in South Africa and Malawi were gathered (524 in South Africa and 64 in Malawi). These were stratified by funder and 28 CBOs (24 in South Africa and 4 in Malawi) were randomly selected by a computer-generated random selection process, pro-rated for population size. Children came from 7 provinces of South Africa: 32.9% KwaZulu-Natal (*n* = 274), 22.2% Gauteng (*n* = 185), 18.5% Mpumalanga (*n* = 154), 8.5% Free State (*n* = 79), 8% Western Cape (*n* = 67), 4.8% Limpopo (*n* = 40), and 4.2% Eastern Cape (*n* = 35). Eleven of the South African CBOs were rurally located (46%), two were in semi-urban areas (8%) and 11 were in urban areas (46%). In Malawi, all four CBOs were located in rural areas (100%) in the Central Region.

Consecutive children (*n* = 989) attending the CBO on data collection days were interviewed together with their primary caregiver using mobile phone technology for data capture (Tomlinson et al., 2009). CBO leadership provided detailed information on CBO services and structure. The study was given ethical approval by the University College London ethics board (reference number 1478/002) and the Health Research Ethics Committee at Stellenbosch University (reference number N10/04/112). Data collectors were trained on the use of mobile technology to collect the data, interview caregivers and children, gather growth measurements and administer two standardised cognitive developmental tests.

Baseline data were gathered in 2012; the follow-up data were collected 12–15 months later (2013–2014). Inclusion rate was high with only 0.7% refusal at baseline. At follow-up, 86.3% were traced and available for inclusion. In the current study, 10 participants were excluded because of missing data at baseline, leaving a total sample of 979 participants.

### Measures

Demographic data included carer report on household variables, employment, food security and family illness. Items on food and nutrition and school functioning were from the Child Status Index (CSI; Nyangara, O’Donnell, Murphy, & Nyberg, 2009). Carer-report items on harsh discipline practices were drawn from the Parent–Child Conflict Tactics Scale (Straus, Hamby, Finkelhor, Moore, & Runyan, 1998) and the International Society for the Prevention of Child Abuse and Neglect (ISPCAN) screening tools (Runyan et al., 2009). Community violence was measured using two child-report items on whether they had seen someone being attacked or whether they had ever been attacked outside their home. Children also provided information on their housing situation, whether their biological parents were still alive, whether they care for sick people or younger children and whether they have enough to eat. HIV status for both child and parents was gathered utilising parental report. Educational risk was a composite measure made up of five binary (yes/no) variables: being in the correct class for their age, irregular attendance, being a slow learner, having recently missed more than a week of school and doing worse than most or struggling in school.

Emotional and behavioural problems were measured by carer report using the short Strengths and Difficulties Questionnaire (SDQ; Goodman, 1997), consisting of nine items divided over several subscales on internalising and externalising behavioural problems (scored 0 = not true, 1 = somewhat true, 2 = certainly true or vice-versa for reverse-scored items), with higher scores indicating worse emotional and behavioural problems. Delinquency was measured using the Externalising and Risk Behaviour Domain (Snider & Dawes, 2006), consisting of three items: how often the child had been drunk or high, how often the child had been arrested and how often the child had beaten someone up (scored 0 = never, 1 = once, 2 = twice, 3 = three or four times, 4 = five times or more). Total scores range from 0 to 12, with higher scores reflecting more delinquency. Depression was measured with child report using an adapted version of the Child Depression Inventory (Kovacs, 1992; current *α *= .63), which has been recently used in South Africa (Cluver et al., 2007; Mueller, Alie, Jonas, Brown, & Sherr, 2011). This scale comprises nine items (scored 0–2, 2 being the worst outcome), with a total score of 0–18 and higher scores indicating worse depression. One of these nine items was used to measure suicidal ideation, which was converted into a binary (yes/no) variable. Self-esteem was measured using the Rosenberg Self-Esteem Scale (current *α *= .60), a child-report ten-item scale with extensive validity and reliability data (Bagley, Bolitho, & Bertrand, 1997; Gray-Little, Williams, & Hancock, 1997; Griffiths et al., 1999; Rosenberg, 1965). Each item is scored 0–3 (3 being the best outcome), with higher scores indicating better self-esteem (total score of 0–30). Post traumatic stress disorder symptomatology was assessed with the ten-item child-report Trauma Symptom Checklist for Children (TSCC; Briere, 1996; current *α *= .70). The items are scored 0 = never, 1 = sometimes, 2 = lots of times, 3 = all the time, with a total score of 0–30 and higher scores indicating worse trauma. Stigma was measured by child report, using the Experience of Stigma, Discrimination and Social Exclusion Domain (Snider & Dawes, 2006), with four items added (current *α *= .74). Quality of life was measured using a short version of the carer-report Paediatric Quality of Life Inventory (PedsQL 4.0; Varni, Seid, & Kurtin, 2001), consisting of 15 items divided over several subscales (scored 0 = never to 4 = almost always): educational, physical, emotional and social functioning. The scores of every subscale were converted into a standardised score from 0 to 100, with higher scores being a sign of better functioning in these subscales. The mean of all subscales was taken to determine total quality of life. Cognitive development was assessed with two age-appropriate developmental tests: a digit span test for working memory (Wechsler, 2004) and the draw-a-person test for general cognitive ability (Goodenough, 1926; Harris, 1963). Developmental disability was measured using the carer-report Ten Questions disability questionnaire (Belmont, 1984).

### Statistical analysis

All analyses were run using SPSS v23 (IBM Corp., 2014). We conducted *t*-tests and *χ* ^2^-tests to identify baseline differences between boys and girls and further explored baseline differences using multiple linear regression analyses. We completed mediation analyses using the SPSS macro PROCESS (Hayes, 2013) and analysed changes over time using repeated measures ANOVAs. In all regression analyses, we controlled for the covariates digit span, draw-a-person test score, any developmental disability, school attendance and caregiver HIV status. The outcomes of interest spanned a range of developmental outcomes (i.e., physical development, behavioural problems, quality of life, educational outcomes, delinquency, psychological well-being and cognitive abilities). Potential moderators were factors in the home that might differ by gender (i.e., exposure to violence, socio-economic status and caring for young or elderly individuals).

## Results

### Sample characteristics

Table 1 shows the baseline demographic variables from the sample according to gender. The study population comprised 476 boys and 503 girls (aged 4–13 years, with *M* = 8.91 and SD = 2.84). There were no significant differences according to gender in terms of country, child age, school attendance, child home, parental bereavement, or HIV status. There were 66 boys (13.9%) and 69 girls (13.7%) who were HIV positive, while 410 boys (86.1%) and 434 girls (86.3%) were not. HIV status did not differ significantly by gender (*χ* ^2^(1) = 0.004, *p *= .95). In terms of bereavement, 70 boys (17.1%) and 80 girls (18.3%) had experienced the death of a mother, 76 boys (18.6%) and 78 girls (17.9%) had experienced the death of a father and 69 boys (16.9%) and 80 girls (18.3%) had experienced the death of both their parents. Overall, 53.4% of children were not orphaned, and bereavement did not differ by gender (*χ* ^2^(4) = 1.25, *p *= .87). School enrolment was high with 96% of the sample enrolled in school including 460 boys (96.6%) and 483 girls (96.0%), which did not differ by gender (*χ* ^2^(1) = 0.26, *p *= .61).

**Table 1.** Baseline differences between boys and girls on demographic and socio-economic variables and cognitive and psychosocial outcomes. Data are Mean (SD) or *N* (%). Difference statistics are *t* (*p*) for continuous variables or *χ* ^2^ (*p*) for categorical variables. Significant differences between genders are in bold type.

	Total	Boys	Girls	Difference statistic
	*N* = 979	*N* = 476	*N* = 503	(*p*-value)
*Demographics*				
Child age	8.91 (2.84)	9.04 (2.82)	8.78 (2.85)	1.40 (.16)
Child HIV positive	135 (13.8%)	66 (13.9%)	69 (13.7%)	0.004 (.95)
Carer gender^a^	930 (95.0%)	451 (94.7%)	479 (95.2%)	0.12 (.73)
Carer age	43.63 (14.97)	44.00 (14.96)	43.27 (14.99)	0.77 (.44)
Carer HIV positive	189 (19.3%)	104 (21.8%)	85 (16.9%)	3.85 (.050)
*Country*				
South Africa	824 (28.4%)	402 (84.5%)	422 (83.9%)	0.057 (.81)
Malawi	155 (15.8%)	74 (15.5%)	81 (16.1%)	
*Parent died*				
Mother	150 (17.8%)	70 (17.1%)	80 (18.3%)	1.25 (.87)
Father	154 (18.2%)	76 (18.6%)	78 (17.9%)	
Both	149 (18.2%)	69 (16.9%)	80 (18.3%)	
None	377 (44.6%)	188 (46.0%)	189 (43.3%)	
Do not know	15 (1.8%)	6 (1.5%)	9 (2.1%)	
Recent bereavement	281 (28.7%)	143 (30.0%)	138 (27.4%)	0.81 (.37)
HIV in household	331 (33.8%)	169 (35.5%)	162 (32.2%)	1.19 (.28)
Family sickness	215 (22.0%)	111 (23.3%)	104 (20.7%)	1.00 (.32)
*Socio-economic variables*				
Informal housing	152 (15.5%)	69 (14.5%)	83 (16.5%)	0.75 (.39)
Household employment	526 (53.7%)	260 (54.6%)	266 (52.9%)	0.30 (.59)
Number of people in household	6.42 (2.90)	6.49 (3.09)	6.35 (2.70)	
Food insecurity	263 (26.9%)	121 (25.4%)	142 (28.2%)	0.98 (.32)
Went to bed hungry last night	127 (13.0%)	55 (11.6%)	72 (14.3%)	1.65 (.20)
Domestic violence score	1.10 (1.61)	1.14 (1.72)	1.05 (1.50)	0.89 (.37)
**Community violence score**	**0.60** (**0.91)**	**0.71** (**0.98)**	**0.50** (**0.82)**	**3.68** (**<.001)**
**Harsh physical discipline score**	**0.57** (**0.68)**	**0.64** (**0.72)**	**0.50** (**0.64)**	**3.17** (**.002)**
Harsh psychological discipline score	0.79 (1.11)	0.80 (1.08)	0.78 (1.14)	0.27 (.79)
Care for younger kids	382 (45.2%)	182 (44.5%)	200 (45.9%)	0.16 (.69)
Care for sick people	343 (40.6%)	156 (38.1%)	187 (42.9%)	1.97 (.16)
Enrolled in school	943 (96.3%)	460 (96.6%)	483 (96.0%)	0.26 (.61)
*Developmental outcomes*				
**Developmental disability**	**441** (**45.0%)**	**230** (**48.3%)**	**211** (**41.9%)**	**4.01** (**.045)**
Stunting	303 (31.7%)	159 (33.9%)	144 (29.5%)	2.13 (.14)
Wasting	31 (3.1%)	13 (2.8%)	17 (3.4%)	0.36 (.55)
Underweight	45 (8.8%)	22 (8.8%)	23 (8.7%)	<0.001 (.98)
**Behavioural problems**	**3.01** (**2.39)**	**3.25** (**2.42)**	**2.78** (**2.33)**	**3.09** (**.002)**
Internalising problems	1.85 (1.56)	1.90 (1.54)	1.81 (1.57)	0.93 (.35)
**Externalising problems**	**1.16** (**1.34)**	**1.35** (**1.44)**	**0.97** (**1.21)**	**4.42** (**<.001)**
Quality of life	91.01 (9.80)	90.52 (9.71)	91.47 (9.88)	1.52 (.13)
Physical functioning	96.51 (10.43)	96.78 (10.21)	96.25 (10.64)	0.78 (.43)
Emotional functioning	89.27 (13.85)	89.67 (13.44)	88.89 (14.24)	0.88 (.38)
Social functioning	89.60 (15.39)	89.16 (15.30)	90.01 (15.48)	0.86 (.39)
**Educational functioning**	**85.36** (**22.06)**	**82.30** (**23.41)**	**88.27** (**20.30)**	**4.17** (**<.001)**
**Number of educational risks**	**0.78** (**1.04)**	**0.93** (**1.14)**	**0.64** (**0.92)**	**4.27** (**<.001)**
**Incorrect class for age**	**272** (**28.8%)**	**156** (**33.9%)**	**116** (**24.0%)**	**11.24** (**.001)**
Irregular school attendance	41 (4.3%)	20 (4.3%)	21 (4.3%)	<0.001 (1.00)
**Slow learner**	**255** (**27.0%)**	**143** (**31.1%)**	**112** (**23.2%)**	**7.45** (**.006)**
**Struggles in school**	**157** (**16.6%)**	**101** (**22.0%)**	**56** (**11.6%)**	**18.23** (**<.001)**
Missed more than a week of school	4 (0.4%)	2 (0.4%)	2 (0.4%)	0.002 (.97)
**Delinquency score**	**0.66** (**1.16)**	**0.80** (**1.29)**	**0.54** (**1.02)**	**3.49** (**.001)**
Depression score	1.08 (1.65)	1.09 (1.65)	1.07 (1.65)	0.24 (.81)
Suicidal ideation	20 (2.0%)	11 (2.3%)	9 (1.8%)	0.33 (.56)
Self-esteem score	20.99 (2.87)	20.90 (2.75)	21.08 (2.97)	0.93 (.35)
Stigma score	0.82 (1.48)	0.85 (1.51)	0.80 (1.45)	0.56 (.57)
Trauma score	3.58 (3.23)	3.53 (3.40)	3.63 (3.06)	0.50 (.62)
Digit span	8.77 (3.96)	8.64 (4.25)	8.90 (3.67)	0.98 (.33)
Draw-a-person score	86.09 (18.57)	85.77 (18.85)	86.38 (18.33)	0.48 (.63)

^a^Number of females.

### Baseline associations between gender and development outcomes

On the outcome measures, boys and girls differed in three main domains: exposure to violence, education outcomes and behavioural problems (see Table 1). Boys experienced more community violence (*M *= 0.71, SD* *= 0.98) than girls (*M *= 0.50, SD* *= 0.82; *t*(923) = 3.68, *p *< .001); and more harsh physical discipline (*M *= 0.64, SD* *= 0.72) than girls (*M *= 0.50, SD* *= 0.64; *t*(977) = 3.17, *p *= .002). Boys were less often in the correct class for their age (66.1%) than girls (76.0%; *χ* ^2^(1) = 11.24, *p = *.001); were more often slow learners (31.1%) than girls (23.2%; *χ* ^2^(1) = 7.45, *p = *.006) and were more likely to struggle in school (22.0% versus 11.6%; *χ* ^2^(1) = 18.23, *p < *.001). Together, this resulted in boys scoring significantly lower on educational functioning (*M *= 82.30, SD* *= 23.41) than girls (*M *= 88.27, SD* *= 20.30; *t*(908) = 4.17, *p *< .001) and suffering a higher number of educational risks (*M *= 0.93, SD* *= 1.14 versus *M *= 0.64, SD* *= 0.92; *t*(864) = 4.27, *p *< .001). In contrast, no differences in cognitive development were found on the digit span test and the draw-a-person test (see Table 1). Boys displayed significantly more behavioural and emotional problems (*M *= 3.25, SD* *= 2.42) than girls (*M *= 2.78, SD* *= 2.33; *t*(977) = 3.09, *p *= .002), and more externalising behavioural problems in particular (*M *= 1.35, *SD *= 1.44 versus *M *= 0.97, SD* *= 1.21); *t*(929) = 4.42, *p *< .001). Boys also scored significantly higher on delinquency measures at baseline (*M *= 0.80, SD* *= 1.29) than girls (*M *= 0.54, SD* *= 1.02; *t*(903) = 3.49, *p *= .001). Finally, boys were rated as exhibiting developmental disability on the Ten Questions disability index more often than girls (48.3% versus 41.9%; *χ* ^2^(1) = 4.01, *p = *.045).

### Baseline associations between gender and educational outcomes

Because initial *t*-tests showed that boys and girls differed strongly on educational outcomes but not on cognitive development outcomes, we ran two multiple linear regression models to see what the unique association between gender and educational outcomes were, controlled for cognitive abilities (digit span score and draw-a-person score), developmental delay (since this differed slightly between the two genders), carer HIV status (because of the marginally significant difference between the two genders) and school attendance (so that any differences would not be due to differing cognitive abilities or lack of attendance). These two adjusted regression analyses showed that being a girl was also associated with significantly better educational functioning (*B* = 5.60; CI = 2.76, 8.44) and with a lower number of educational risks (*B* = −0.22; CI = −0.35, −0.097).

### Baseline mediation effect of gender through violence exposure on school outcomes

Regressional mediation analyses (see Table 2) showed that the association between gender and educational functioning was mediated by exposure to community violence and harsh physical discipline, meaning that being a boy is associated with more exposure to these two types of violence, which in turn is associated with a reduction in educational functioning. A similar mediation effect was found for educational risk: being a boy increases the exposure to community violence and harsh physical discipline, which is associated with more educational risks.

**Table 2.** Regressional mediation analyses showing the direct effect of gender (1 = boy, 2 = girl) on educational functioning and educational risk, and the indirect effect of gender through exposure to community violence and harsh physical discipline. All direct effects and indirect effects were significant.

	Educational functioning score	Number of educational risks
	B (CI)	B (CI)
Gender	0.61 (0.27, 0.95)	−0.19 (−0.31, −0.064)
Community violence	−0.18 (−0.39, 0.021)	0.082 (0.0071, 0.16)
Harsh physical discipline	−0.33 (−0.58, −0.06)	0.16 (0.066, 0.26)
Carer HIV status	−0.032 (−0.48, 0.42)	0.082 (−0.083, 0.25)
Digit span	0.059 (0.013, 0.10)	−0.062 (−0.078, −0.045)
Draw-a-person score	−0.0086 (−0.018, 0.0009)	−0.0018 (−0.0053, 0.0017)
Any developmental delay	−1.48 (−1.83, −1.13)	0.57 (0.45, 0.70)
School attendance	−1.20 (−1.92, −0.48)	1.35 (1.09, 1.62)
**Direct effect**	**0.61** (**0.27, 0.95)**	−**0.19** (−**0.31,** −**0.064)**
**Indirect effect: community violence**	**0.032** (**0.0013, 0.096)**	−**0.014** (−**0.040,** −**0.0003)**
**Indirect effect: harsh physical discipline**	**0.037** (**0.0060, 0.96)**	−**0.018** (−**0.041,** −**0.0030)**
*R^2^*	*0.16*	*0.31*

Notes: CI = confidence interval. Reported model fit for the logistic regression model (correct class for age) is the McFadden *R^2^*.

### Baseline mediation effect of violence exposure through behavioural problems on school outcomes

Additional mediation analyses (see Table 3) showed a consistent indirect effect of both exposure to community violence and harsh physical punishment on educational functioning and number of educational risks through general behavioural problems and delinquency. This indicates that both types of violence increase behavioural problems and delinquency, which both in turn decrease educational functioning and increase the number of educational risks. Externalising problems did not have a mediating effect on any of the educational outcomes.

**Table 3.** Regressional mediation analyses showing the direct effects of exposure to community violence and harsh physical discipline on educational functioning and educational risk, and the indirect effects of the two types of violence through (externalising) behavioural problems and delinquency. Significant effects are in bold type.

	Exposure to community violence		Harsh physical discipline	
	Educational functioning score	Number of educational risks	Educational functioning score	Number of educational risks
	B (CI)	B (CI)	B (CI)	B (CI)
Community violence	−0.12 (−0.33, 0.085)	0.052 (−0.023, 0.13)	–	–
Harsh physical discipline	–	–	−0.14 (−0.41, 0.13)	0.099 (−0.0003, 0.20)
Externalising problems	−0.080 (−0.28, 0.12)	−0.029 (−0.10, 0.046)	−0.060 (−0.26, 0.14)	−0.033 (−0.11, 0.042)
General behavioural problems	**−0.17(−0.28, −0.052)**	**0.082(0.040, 0.12)**	**−0.17(−0.29, −0.059)**	**0.080(0.038, 0.12)**
Delinquency score	**−0.17(−0.32, −0.020)**	**0.095(0.039, 0.12)**	**−0.17(−0.32, −0.020)**	**0.093(0.037, 0.15)**
Carer HIV status	−0.057 (−0.50, 0.39)	0.090 (−0.073, 0.25)	−0.049 (−0.49, 0.40)	0.096 (−0.066, 0.26)
Digit span	**0.074(0.028, 0.12)**	**−0.065(−0.081, −0.048)**	**0.075(0.031, 0.12)**	**−0.068(−0.085, −0.052)**
Draw-a-person score	−0.007 (−0.017, 0.002)	−0.002 (−0.005, 0.001)	−0.007 (−0.016, 0.0027)	−0.002 (−0.006, 0.001)
Any developmental delay	**−1.38(−1.73, −1.03)**	**0.54(0.41, 0.67)**	**−1.35(−1.71, −1.00)**	**0.53(0.40, 0.66)**
School attendance	**−1.03(−1.75, −0.31)**	**1.31(1.04, 1.57)**	**−1.05(−1.77, −0.33)**	**1.32(1.05, 1.58)**
*Direct effect*	−*0.12* (−*0.33, 0.085)*	*0.052* (−*0.023, 0.13)*	−*0.14* (−*0.41, 0.13)*	*0.099* (−*0.0003, 0.20)*
*Indirect effect : externalising problems*	−*0.014* (−*0.064, 0.022)*	−*0.005* (−*0.025, 0.008)*	−*0.030* (−*0.15, 0.068)*	−*0.017* (−*0.064, 0.024)*
*Indirect effect: general behavioural problems*	**−*0.071*(−*0.15,* −*0.016)***	***0.035*(*0.016, 0.069)***	**−*0.15*(−*0.28,* −*0.039)***	***0.070*(*0.031, 0.12)***
*Indirect effect: delinquency*	**−*0.049*(−*0.11,* −*0.007)***	***0.027*(*0.008, 0.050)***	**−*0.07*(−*0.16,* −*0.011)***	***0.038*(*0.012, 0.071)***
*R^2^*	*0.18*	*0.32*	*0.18*	*0.33*

Notes: CI = confidence interval.

### Follow-up

A total of 146 (14.8%) children were lost to follow-up. Children lost to follow-up had significantly younger caregivers (*t*(977) = 3.66, *p *< .001), scored higher on perceived stigma (*t*(136) = 3.05, *p *= .003), lived more in South Africa (*χ* ^2^(1) = 8.87, *p = *.003), more often lived in a shack (*χ* ^2^(1) = 12.61, *p < *.001), were more often not enrolled in school (*χ* ^2^(1) = 4.88, *p = *.027) and were more often food insecure (*χ* ^2^(1) = 5.16, *p = *.023). There were no gender differences in loss to follow-up.

As can be seen in Table 3, all the gender differences found at baseline were still significant at follow-up. In addition, at follow-up quality of life also differed significantly by gender (*t*(831) = 2.51, *p *= .012), with boys having a lower average quality of life (*M *= 92.69, SD* *= 8.36) than girls (*M *= 94.03, SD* *= 7.06).

Repeated measures analyses (not controlling for covariates) did not show any differences in change over time on educational, cognitive and psychosocial outcomes according to gender (see Table 4). However, a main effect of time on harsh physical discipline was found (*F*(1, 831) = 25.94, *p* < .001), indicating that overall harsh physical discipline had reduced from baseline to follow-up. There was no significant main effect of time on community violence scores (*F*(1, 818) = 0.038, *p* = .85) (Table 5) .

**Table 4.** Child outcomes by gender at follow-up (*N* = 833). Data are Mean (SD) or *N* (%). Difference statistics are *t* (p) for continuous outcomes and *χ* ^2^ (*p*) for categorical outcomes.

	Boys (*n* = 401)	Girls (*n* = 432)	Difference statistic (*p*-value)
Depression score	0.86 (1.57)	0.75 (1.37)	0.99 (.32)
Trauma score	4.06 (3.77)	4.25 (3.50)	0.75 (.45)
Self-esteem score	22.12 (3.85)	22.31 (3.81)	0.70 (.49)
Behavioural and emotional problems	2.97 (2.61)	2.74 (2.28)	1.35 (.18)
Internalising problems	1.66 (1.73)	1.74 (1.61)	0.71 (.48)
**Externalising problems**	**1.31** (**1.43)**	**1.00** (**1.29)**	**3.32** (**.001)**
**Quality of life**	**92.69** (**8.36)**	**94.03** (**7.06)**	**2.51** (**.012)**
**Delinquency score**	**0.68** (**1.25)**	**0.49** (**1.07)**	**2.36** (**.018)**
**Number of educational risks**	**1.01** (**1.18)**	**0.62** (**0.95)**	**5.10** (**<.001)**
Digit span	8.90 (3.65)	9.10 (3.41)	0.79 (.43)
Draw-a-person score	90.74 (17.98)	91.81 (16.62)	0.89 (.38)
Stigma score	0.91 (1.45)	0.91 (1.42)	0.085 (.93)
Domestic violence score	0.92 (1.51)	0.80 (1.25)	1.30 (.19)
**Community violence score**	**0.68** (**0.85)**	**0.55** (**0.75)**	**2.22** (**.027)**
**Harsh physical discipline**	**0.48** (**0.66)**	**0.38** (**0.57)**	**2.30** (**.022)**
Harsh psychological discipline	0.67 (0.97)	0.62 (0.96)	0.75 (.45)

Notes: The bold typed variables and data differ significantly between genders.

**Table 5.** Change in child outcomes over time by gender (*N* = 823). Data are Mean (SD), difference statistic is *F*(*p*). Repeated measures ANOVA analyses; effect of time * child gender. None of the interaction effects were significant.

	Boys (*n* = 393)		Girls (*n* = 430)		
	Baseline	Follow-up	Baseline	Follow-up	Difference statistic (*p*-value)
Depression score	1.07 (1.63)	0.86 (1.57)	1.01 (1.59)	0.76 (1.38)	0.073 (.79)
Trauma score	3.51 (3.45)	4.07 (3.78)	3.64 (2.95)	4.28 (3.50)	0.083 (.77)
Self-esteem score	20.94 (2.76)	22.23 (3.80)	21.19 (2.88)	22.33 (3.82)	0.21 (.65)
Behavioural and emotional problems	3.26 (2.44)	2.97 (2.61)	2.75 (2.29)	2.74 (2.28)	2.00 (.16)
Internalising problems	1.91 (1.56)	1.66 (1.73)	1.80 (1.51)	1.74 (1.61)	1.88 (.17)
Externalising problems	1.35 (1.43)	1.31 (1.43)	0.94 (1.20)	1.00 (1.29)	0.70 (.40)
Quality of life	90.53 (9.76)	92.69 (8.36)	91.77 (9.80)	94.03 (7.06)	0.016 (.90)
Number of educational risks	0.92 (1.13)	1.02 (1.18)	0.63 (0.91)	0.63 (0.96)	1.61 (.21)
Digit span	8.63 (4.25)	8.92 (3.65)	8.86 (3.71)	9.09 (3.43)	0.052 (.82)
Draw-a-person score	86.20 (19.09)	91.12 (17.91)	86.20 (18.18)	91.65 (16.54)	0.12 (.73)
Stigma score	0.73 (1.35)	0.89 (1.44)	0.74 (1.35)	0.91 (1.39)	0.031 (.86)
Domestic violence score	1.22 (1.78)	0.93 (1.51)	0.99 (1.38)	0.80 (1.25)	0.56 (.45)
Community violence score	0.72 (1.00)	0.68 (0.85)	0.49 (0.78)	0.55 (0.75)	1.78 (.18)
Harsh physical discipline	0.65 (0.72)	0.48 (0.66)	0.50 (0.64)	0.38 (0.57)	0.67 (.41)
Harsh psychological discipline	0.82 (1.11)	0.67 (0.97)	0.78 (1.14)	0.62 (0.96)	0.030 (.86)

## Discussion

In this age range (4–13 years), there were no gender differences on basic demographic factors with the boys and girls coming from very similar backgrounds. However, at baseline, boys were worse off in a number of domains. Boys were more likely to be the recipient of harsh punishments, more likely to be exposed to community violence and more likely to have behavioural problems. In addition, boys were doing significantly worse at school than girls.

Overall school enrolment was high, as 96% of our sample enrolled in school, reflecting the universal provision of primary school in both South Africa and Malawi. However, school access is only the first step in educational achievement. Attendance, school performance and being in the correct class for one’s age are additional factors of importance. The baseline data clearly show boys performing significantly lower on these more detailed educational measures. It may be possible that a number of girl-targeted initiatives focus specifically on educational access for girls, and as such boys may be overlooked or under-recognised in terms of educational need.

At follow-up, the educational risk for boys persisted. As educational risk factors may well be precursors to HIV risk behaviour (Orkin, Boyes, Cluver, & Zhang, 2014) in older children, it seems important to ensure equity of education in all domains for both girls and boys. Educational risk has a number of long-term effects on life trajectories (Doyle, Mavedzenge, Plummer, & Ross, 2012). Those who perform poorly are more likely to drop out of school. Educational achievement is associated with adult earnings, and good educational achievement is a pathway to employment and a means out of poverty. Furthermore, disengagement in education may be related to more risk activity, where boys who are bored or not engaged in education may move into gang activity, violence and be more likely to pick up HIV risk behaviours including multiple sexual partners, earlier sexual debut (Peltzer, 2010), alcohol consumption and teenage pregnancy. Clearly, attention to boys is important with specific needs to promote educational access, attendance and achievement. There are currently a number of initiatives to promote and support educational engagement for girls. Our data suggest that initiatives for boys should also be considered.

At follow-up, boys also recorded significantly poorer quality of life than girls. These data would suggest that at younger ages, where universal primary school access is available, girls and boys are similar in many aspects of their functioning, including levels of depression, trauma and behaviour. However, on a number of measures, boys are scoring significantly lower and girls are scoring significantly higher. Equity may thus be a guiding principle to examine provision.

Our analysis of change scores, which shows whether there is a difference in change over time according to gender, showed no significant results. On the one hand, this is reassuring as it suggests that there is no variation in provision according to gender in terms of CBO interventions over time. If there were any gender skews, this would be reflected in a disproportionate achievement or provision, which was not seen. On the other hand, it also means that the gap between boys and girls seen at baseline in educational performance is not being closed. It might be necessary for boys to get more educational support than girls from the CBOs, which is not currently happening and is thus a point of potential future improvement and need.

These data show the importance of tracking gender differences at baseline and follow-up and examining provision according to gender in order to explore any systematic variations. They also point to the ramifications of violence in the community. While CBOs may not be able to change community violence as this is outside their zone of influence, they are particularly well-placed to address the use of physical violence as a form of discipline, as children attend the CBOs together with their caregivers. Indeed, this study did show an overall decrease in harsh physical discipline over time, which may partly have been linked to CBO input directly with parenting programmes, or indirectly by alleviating some of the harsh living conditions associated with violence. In addition, boys showed specific inequity in educational risk which should be addressed at an early age if it is not to translate into longer term educational difficulties. Studies show that exposure to family and community violence affects child coping and mental health (Mohammad, Shapiro, Wainwright, & Carter, 2015). This study uniquely contributes to the literature because, although some gender-specific studies have been carried out on, for instance, the biological response to HIV, very few studies – if any – exist that longitudinally tracked developmental outcomes of children by gender in this area and that look at the mediating effects of violence in particular. As the study was conducted within a CBO context, it allowed for some unique insights into the services provided to children by the community, and whether this service provision differed according to gender.

Limitations of the study include the fact that all participants were attending CBOs with no control or comparison group. Families may have self-selected into receiving CBO services. Yet it does provide a comprehensive view of child functioning in high HIV-affected areas. Given the solid response rate and the high follow-up rate, these data suggest a need not only to examine gender provision generally, but also to focus specifically on the needs of boys. Despite the improvements seen over time, boys still experienced significantly more harsh physical discipline than girls, and had elevated and persisting educational risks. Interventions might have to be focused more specifically on boys in order to close the equity gap.
